# Function-Related Positioning of the Type II Secretion ATPase of *Xanthomonas campestris* pv. campestris

**DOI:** 10.1371/journal.pone.0059123

**Published:** 2013-03-11

**Authors:** Yih-Lin Chen, Nien-Tai Hu

**Affiliations:** Institute of Biochemistry, National Chung Hsing University, Taichung, Taiwan, Republic of China; Centre National de la Recherche Scientifique, Aix-Marseille Université, France

## Abstract

Gram-negative bacteria use the type II secretion (T2S) system to secrete exoproteins for attacking animal or plant cells or to obtain nutrients from the environment. The system is unique in helping folded proteins traverse the outer membrane. The secretion machine comprises multiple proteins spanning the cell envelope and a cytoplasmic ATPase. Activity of the ATPase, when copurified with the cytoplasmic domain of an interactive ATPase partner, is stimulated by an acidic phospholipid, suggesting the membrane-associated ATPase is actively engaged in secretion. How the stimulated ATPase activity is terminated when secretion is complete is unclear. We fused the T2S ATPase of *Xanthomonas campestris* pv. campestris, the causal agent of black rot in the crucifers, with fluorescent protein and found that the ATPase in secretion-proficient cells was mainly diffused in cytoplasm. Focal spots at the cell periphery were detectable only in a few cells. The discrete foci were augmented in abundance and intensity when the secretion channel was depleted and the exoprotein overproduced. The foci abundance was inversely related to secretion efficiency of the secretion channel. Restored function of the secretion channel paralleled reduced ATPase foci abundance. The ATPase foci colocalized with the secretion channel. The ATPase may be transiently associated with the T2S machine by alternating between a cytoplasmic and a machine-associated state in a secretion-dependent manner. This provides a logical means for terminating the ATPase activity when secretion is completed. Function-related dynamic assembly may be the essence of the T2S machine.

## Introduction

Pathogenic bacteria use the type II secretion (T2S) system to attack host cells and environmental bacteria use it to promote growth in their niches [1]. Although the system is involved in exoprotein translocation across the outer membrane of Gram-negative bacteria, it comprises proteins extending over the cell envelope. These proteins include an outer membrane protein; 5 type IV pilin-like proteins, named pseudopilins; and 4 cytoplasmic membrane proteins [2]. Moreover, an essential ATPase is predicted to be located in cytoplasm, a compartment separated by the cytoplasmic membrane from where the exoprotein is located before its export.

The outer membrane protein named secretin (D protein) assembles as a ring-like dodecamer to form the secretion channel whose passage is usually blocked by a gate-like structure [3], [4]. Presumably, the channel is opened passively only when exoprotein secretion is in demand. Inferred by analogy to the type IV pilus (T4p) biogenesis system, the T2S ATPase has been proposed to drive the assembly of pseudopilins into pseudopilus, which in turn pushes the exoprotein through the secretion channel [5]. Consistent with the hypothesis, interaction between exoprotein and the periplasmic domain of secretin suggested that, before its secretion, the exoprotein may be situated in a cavity at the entrance end of the secretion channel constituted by the periplasmic domain of secretin [4], [6], [7]. Interaction between exoprotein and subassembly of pseudopilins further implied that the exoprotein may also interact with the tip of pseudopilus [6].

Pseudopilus assembled from the major pseudopilin (G protein) is a filamentous structure similar to T4p, as suggested by molecular modeling with crystallography and electron microscopy [8]. T4p is the long, thin filament on the cell surface of eubacteria and archaea involved in bacterial motility, cell adhesion, DNA uptake and biofilm formation. Surface-exposed pseudopili are observed when the major pseudopilin is expressed at a high level [9], [10]. Structural analysis revealed that the filament is held together by extensive hydrophobic interactions among the N-terminal α-helices of the type IV pilins or the major pseudopilins [5], [8]. The spiraling helix bundle provides the strength and flexibility required for various biological functions of the filamentous structure. The major pseudopilin is targeted to the cytoplasmic membrane independent of the T2S system [11], [12]. Its N-terminal hydrophobic segment is anchored in the membrane and its C-terminal domain in the periplasm. Little is known about how the pilins or the major pseudopilins prepositioned in the cytoplasmic membrane are assembled into the growing filament. The cytoplasmic ATPase recruited to the membrane may provide the driving force. A pseudopilus tip complex of 3 minor pseudopilins (I, J, K proteins) was revealed from its crystal structure [13]. Genetic studies and molecular modeling further suggested that the tip complex may self-assemble and prime the pseudopilus elongation [14].

The T2S ATPase belongs to the secretion ATPase superfamily, whose members also include ATPases required for extension and retraction of the T4p, type IV secretion and archaeal flagellar assembly [15]. Although recognized primarily as a cytoplasmic protein, T2S ATPase could associate with the membrane by direct interaction with the bitopic cytoplasmic membrane protein L in an ATP binding-stimulated manner [16], [17]. ATP hydrolysis activity of the ATPase is essential for secretion but not for membrane association [17]. *In vitro* ATPase activity of the *Vibrio cholerae* T2S ATPase was stimulated by the acidic phospholipid cardiolipin only when copurified with the cytoplasmic domain of the membrane protein L [18]. This observation suggests that stable association of the T2S ATPase with the cytoplasmic membrane protein L is a prerequisite for activation by the membrane. It also implies that the ATPase, when associated with the secretion machine, may be activated for engagement in the secretion process. However, whether the ATPase remains activated when its task is accomplished is unknown. Alternatively, it may revert to its inactive state through an unknown mechanism.

We aimed to decipher how the T2S ATPase works in coordination with the rest of the secretion machine. Specifically, we addressed how the ATPase prevents itself from being activated after secretion is complete. We hypothesized that the ATPase may cycle between a cytoplasmic and membrane-associated state in a secretion-dependent manner. By fusing a fluorescent protein to the T2S ATPase of *Xanthomonas campestris* pv. campestris, the causal agent of black rot in the crucifers, we observed in intact cells that the ATPase appeared as focused spots at the cell periphery if the exoprotein was kept from being secreted and diffused in cytoplasm as exoprotein was secreted. It alternates between a cytoplasmic and machine-associated state that is tightly coupled to the secretion process. As exoprotein stalls in the periplasm, the ATPase stays associated with the membrane-bound machine and disperses to the cytoplasm when exoprotein secretion is achieved.

## Results

### XpsE-ECFP in secretion-proficient cells was diffused in cytoplasm, with few cells showing focused spots at the cell periphery

**Figure 1:**
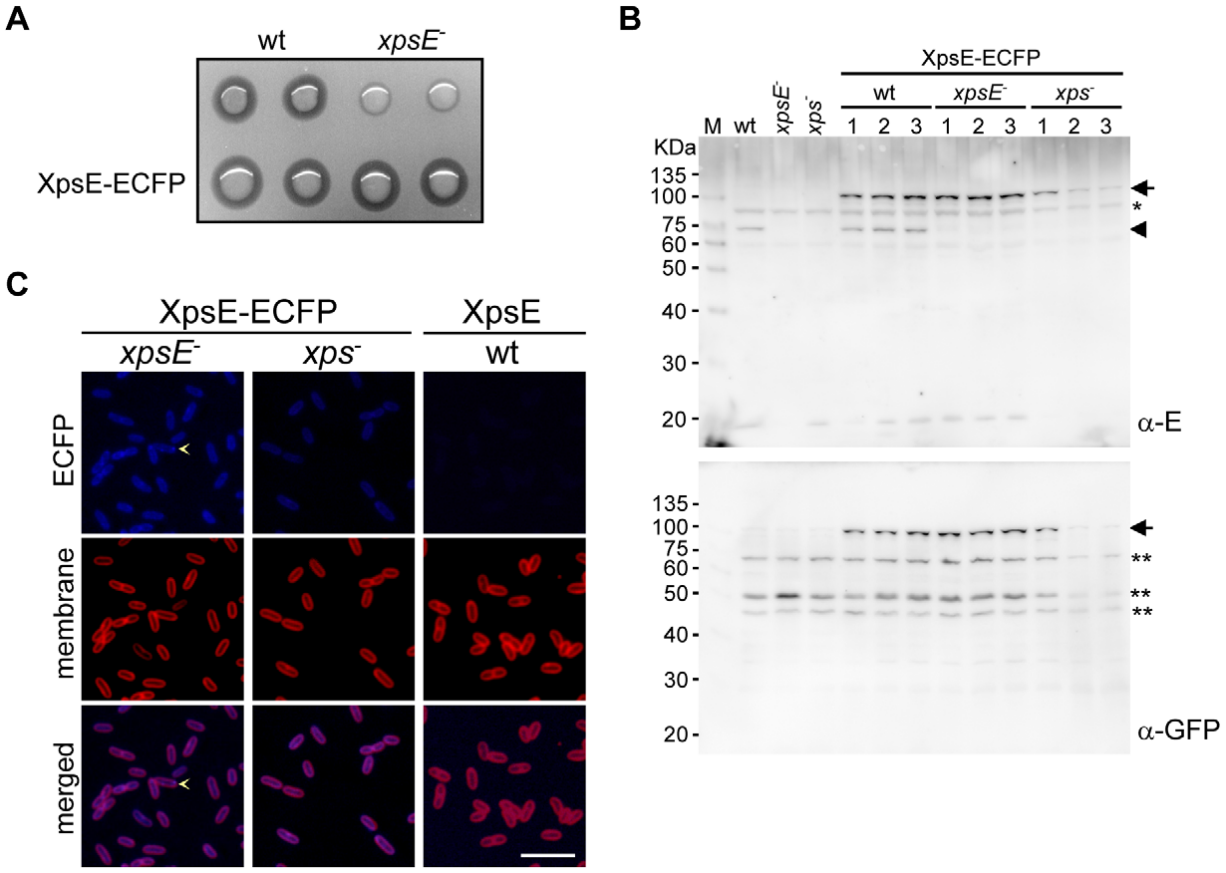
Fluorescence microscopy of the plasmid-encoded XpsE-ECFP complementing the *xpsE*-null strain. (A) Starch plate assay for α-amylase secretion. The parental strain (XC1701, designated as wt) and the *xpsE*-null strain (XC1723, designated as *xpsE* ^−^) are positive and negative controls, respectively. XpsE-enhanced cyan fluorescent protein (ECFP) represents the presence of a plasmid carrying an *xpsE-ecfp* gene. (B) Immunoblot analysis of protein abundance of plasmid-encoded XpsE-ECFP in the parental (wt), the *xpsE*-null (*xpsE* ^−^) strain or the strain lacking all *xps* genes (*xps* ^−^) as detected by anti-XpsE antiserum (top panel), and its stability, as detected by anti-GFP antiserum (bottom panel). The numbers depict different transformants. The plasmid-encoded XpsE-ECFP is indicated by an arrow and the chromosomal XpsE by an arrowhead. * and **, cross-reactive band in the *X. campestris* pv. campestri cell lysate interacting with anti-XpsE and anti-GFP antisera, respectively. (C) Fluorescence microscopy images of the plasmid-encoded XpsE-ECFP in the *xpsE*-null (*xpsE* ^−^) strain or the strain lacking all *xps* genes (*xps* ^−^). In both cases, the transformant labeled #1 was examined. The parental strain (wt) expressing the XpsE devoid of ECFP is included as a negative control. Top: visualized for ECFP; middle: visualized for the fluorescent membrane dye FM4-64; bottom: merged fluorescent images of ECFP and FM4-64. XpsE-ECFP foci appearing at the cell periphery are indicated by an arrowhead. Scale bar, 5 µm.

To determine whether the T2S ATPase cycles between a cytoplasmic and a membrane-associated state in a secretion-related manner, we first examined the subcellular distribution of T2S ATPase in intact cells by tagging the T2S ATPase of *Xanthomonas campestris* pv. campestris XpsE at its C terminus with enhanced cyan fluorescent protein (ECFP). The XpsE-ECFP complemented the *xpsE*-null strain (XC1723) (Table S1) and restored α-amylase secretion (Figure 1A), which suggests that the XpsE-ECFP fusion protein was functionally intact. Immunoblot analysis revealed that the plasmid-encoded XpsE-ECFP was structurally intact (Figure 1B). In contrast to the membrane-specific fluorescence, the ECFP fluorescence in most cells appeared diffuse in cytoplasm (Figure 1C). Only one or two focused fluorescent spots per cell were detected at the cell periphery in a few cells. Although polar distribution of fluorescent foci appeared to be predominant, nonpolar ones were also observed.

In the merged image, the ECFP image displayed not only the cytoplasmic and focal appearing fluorescence but also a membrane-associated signal. The latter may be due to the intrinsic property of the T2S ATPase, which may make the XpsE-ECFP tend to bind peripherally to the membrane all around the cell. In consistent with the suggestion, similar membrane-associated signal all around the cell was detectable in the merged image of the XpsE-ECFP expressed in the mutant strain XC17433, which lacks all components of the T2S machine (Figure 1C).

To confirm that the fluorescence arose from the XpsE-ECFP, we collected fluorescence image of the parental strain (XC1701) devoid of ECFP in the same manner as those of the strain complemented with XpsE-ECFP. The results (labeled as wt in Figure 1C) indicated the parental strain displayed fluorescence signal that was barely detectable.

### Foci abundance and fluorescence intensity of XpsE-ECFP augmented by depleting secretin and overproducing α-amylase

**Figure 2:**
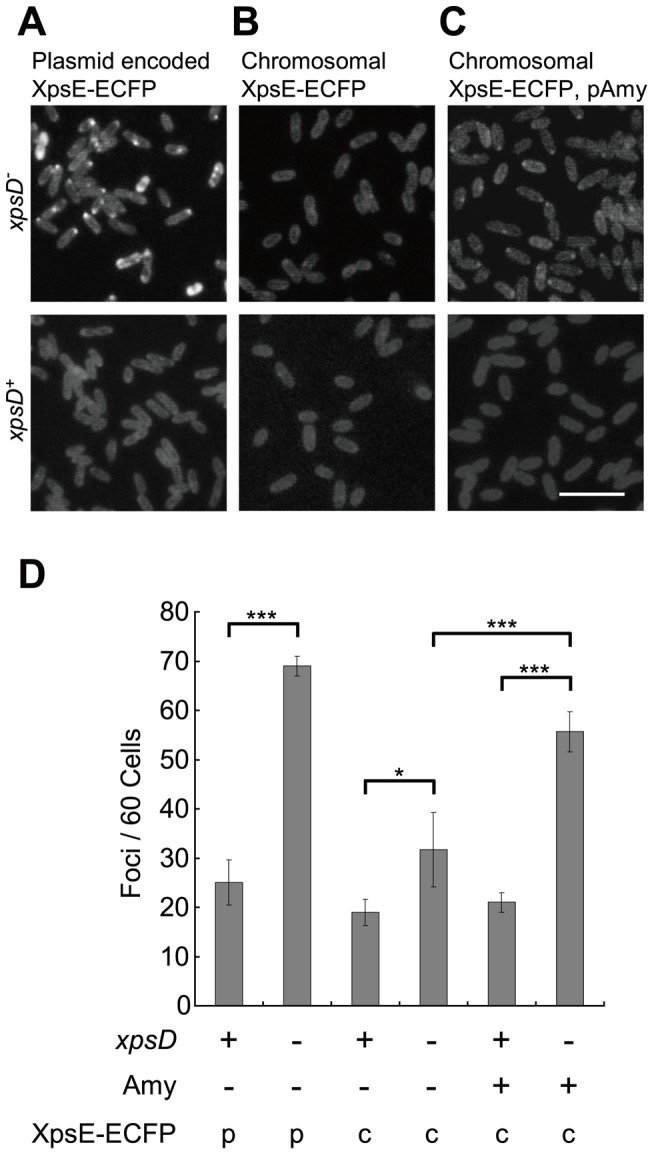
Augmentation of XpsE-ECFP foci abundance and intensity by depleting the *xpsD* gene and overexpressing α-amylase. Fluorescence microscopy of (A) plasmid-encoded XpsE-ECFP in *xpsD* ^−^ (XC1708, top) or *xpsD* ^+^ (XC1701, bottom), (B) chromosome-encoded XpsE-ECFP in *xpsD* ^−^ (XC1753, top) or *xpsD* ^+^ (XC1751, bottom) strain, and (C) chromosome-encoded XpsE-ECFP in *xpsD* ^−^ (XC1753, top) or *xpsD* ^+^ (XC1751, bottom) strain, each supplemented with the plasmid pAmy encoding the full-length α-amylase. Scale bar, 5 µm. (D) Quantitative analysis of foci abundance by plasmid-encoded XpsE-ECFP (p) or chromosome-encoded XpsE-ECFP (c) in the genetic background of *xpsD* ^+^ or *xpsD* ^−^, with or without overexpressed α-amylase (Amy). Data are mean foci counts per 60 cells from 3 independent fields. * P = 0.052; *** P<0.001.

If the focal-appearing XpsE-ECFP at the cell periphery corresponds to the membrane-associated XpsE, it would be scarce in a secretion-competent strain, given the efficient turnover of the secretion process. Thus, blockage of secretion at a late stage should increase the abundance of focal-appearing XpsE-ECFP. We introduced a plasmid encoding XpsE-ECFP into the *xpsD*-null strain (XC1708) (Table S1) deficient in T2S because of lack of secretion channel. For comparison, the same plasmid was introduced into the *xpsD*-plus strain (XC1701) (Table S1). Congruent with our prediction, the number of cells showing focal-appearing XpsE-ECFP was greater with the genetic background of the *xpsD*-null than *xpsD*-plus strain (Figures 2A and 2D).

To rule out the possibility that the focal appearing XpsE-ECFP may have arisen from protein aggregation, we replaced the chromosomal *xpsE* gene with *xpsE-ecfp* in both the *xpsD*-null and *xpsD*-plus strains by a two-step recombination procedure [19]. The chromosome-encoded XpsE-ECFP was functional in α-amylase secretion (Figure S1A). Despite weakened fluorescence intensity and slightly reduced protein abundance, the augmented foci formation in the *xpsD*-null strain remained statistically significant (Figures 2B, 2D and S1B). To compensate for the weakened effect caused by integrating *xpsE-ecfp* in chromosomes, we overproduced α-amylase by introducing an extra copy of the *amy* gene carried on a pAmy plasmid because the *xpsD* gene disruption causes secreted substrates to accumulate in the periplasm [20]. As anticipated, overexpression of the full-length α-amylase encoded by pAmy augmented fluorescence intensity and the abundance of the foci formed by the chromosome-encoded XpsE-ECFP only in the *xpsD*-null strain (XC1753)(Table S1; Figures 2C and 2D). Because α-amylase is targeted to the periplasm by its N-terminal signal peptide, its translocation to periplasm is blocked by depletion of the signal peptide. Introduction of the plasmid encoding the α-amylase lacking its N-terminal signal peptide (cAmy) increased the abundance of XpsE-ECFP foci but at a level similar to that of the control supplemented with an empty vector (Figure S2B). Both levels were less than that with overexpressed full-length α-amylase. In addition, fluorescence of the discrete foci was greater with the full-length than truncated α-amylase (Figure S2A). In contrast, overexpression of the periplasmic maltose binding protein (MBP) did not increase foci abundance. Instead, its overproduction appeared to have a slight negative effect on XpsE-ECFP foci formation in the *xpsD*-null strain, for unclear reasons. Regardless, these findings indicated that fluorescence enhancement is specifically induced by periplasmic accumulation of the exoprotein.

**Figure 3:**
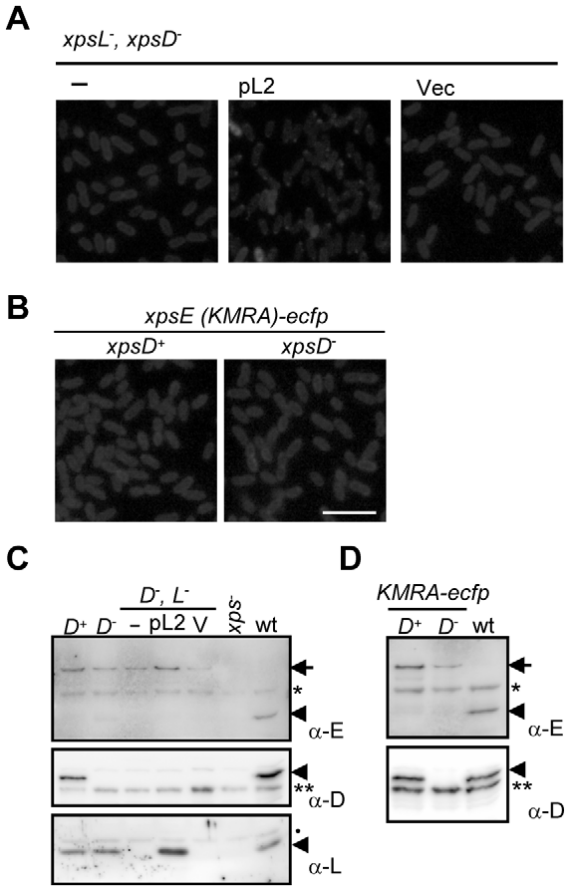
Chromosome-encoded XpsE-ECFP foci formation in the *xpsD* ^−^ background reliant on XpsL and ATP-binding to XpsE. (A) Fluorescence microscopy of chromosome-encoded XpsE-ECFP in *xpsD* ^−^, *xpsL* ^−^ strain (XC1760, labeled as ‘−’) or supplemented with the pCPP30-based plasmid pL2 (labeled as ‘pL2’) encoding the wild-type XpsL or an empty vector (labeled as ‘Vec’). (B) Fluorescence microscopy of chromosome-encoded, double mutated XpsE-ECFP (K331M and R504A, designated KMRA) in *xpsD* ^+^ (XC1757, left) or *xpsD* ^−^ (XC1758, right). The plasmid pAmy encoding the full length α-amylase was included in all strains. Scale bar, 5 µm. (C) Immunoblot of XpsE-ECFP (top panel), XpsD (middle panel) and XpsL (bottom panel). ‘*D* ^+^’ designates the *xpsD*-plus strain with an integrated *xpsE-ecfp* gene (XC1751). ‘*D* ^−^’ designates the *xpsD*-null strain with an integrated *xpsE-ecfp* gene (XC1753). ‘*D* ^−^, *L* ^−^’ designates the *xpsD*/*xpsL* double knockout strain with an integrated *xpsE-ecfp* gene (XC1760). ‘*xps* ^−^’ designates the mutant strain lacking all components of the T2S machine with an integrated *xpsE-ecfp* gene (XC17433). ‘wt’ designates the parental strain (XC1701) expressing the wild-type XpsE without fusing to ECFP. ‘−’ designates the absence of additional plasmid; ‘pL2’ and ‘V’ designate, respectively, the presence of the plasmid pL2 and an empty vector, (D) Immunoblot of chromosome-encoded double mutated XpsE(KMRA)-ECFP in the *xpsD*-plus (designated as ‘D^+^’) and the *xpsD*-null (designated as ‘D^−^’) strain. ‘wt’ designates the parental strain (XC1701) expressing the wild-type XpsE without fusing to ECFP. XpsE-ECFP is pointed by an arrow. XpsE, XpsD and XpsL are each pointed by an arrowhead in separate immunoblot. *, ** and • represent cross-reactive band in the *X. campestris* pv. campestri cell lysate interacting with anti-XpsE, anti-XpsD and anti-XpsL antisera, respectively.

In addition, XpsE-ECFP foci did not form in the *xpsD*/*xpsL* double-knockout mutant but reappeared with introduction of a plasmid encoding XpsL (Figure 3A). The XpsE-ECFP protein level was equivalent in the *xpsD*-null and the *xpsD*/*xpsL* double-knockout mutant, although introduction of the plasmid pL2 into the double-knockout mutant did raise protein level of both XpsE-ECFP and XpsL (Figure 3C). Furthermore, the ATP binding-deficient mutant XpsE(K331M, R504A), when expressed as a chromosome-encoded ECFP fusion protein, was diffused in all cells, whether in the *xpsD*-plus or the *xpsD*-null background, in the presence of pAmy (Figure 3B). The XpsE(K331M, R504A) protein abundance appeared to be slightly lower in the *xpsD*-null strain than in the *xpsD*-plus strain (Figure 3D). As predicted, expression of XpsE(K331M, R504A) as plasmid-encoded XpsE-ECFP in the secretion competent strain did not interfere with secretion. Therefore, the observed XpsE-ECFP foci corresponded to a functionally relevant recruitment of the T2S ATPase to the membrane-anchored secretion machine.

To assess if augmentation of the XpsE-ECFP foci in the secretin-depleted strain corresponds to an increase in membrane-associated XpsE-ECFP, we performed subcellular fractionation experiment [17]. However, we were unable to detect more membrane-associated XpsE-ECFP in the *xpsD*-null than in the *xpsD*-plus strain. Possibly, transient association of the focal appearing XpsE-ECFP with membrane is labile to cell disruption during subcellular fractionation. Alternatively, subcellular fractionation may not be as sensitive as fluorescence microscopy to detect the small increase of the XpsE-ECFP as membrane-associated state in the secretin-depleted strain.

Inverse association of XpsE-ECFP foci formation and secretion efficiency of the secretion channel

To further examine the notion that the XpsE-ECFP foci in the *xpsD*-null strain is caused by an impaired secretion process, we predict the number of cells showing focused spots should decrease with restored function of the secretion channel. We attempted to express the secretin XpsD driven by the *E. coli* P_BAD_ promoter, known for its tight regulation [21]. However, the P_BAD_-driven *xpsD* gene was expressed in the amount sufficient to complement the *xpsD*-null mutant in *X. campestris* pv. campestris in the absence of the inducer.

**Figure 4:**
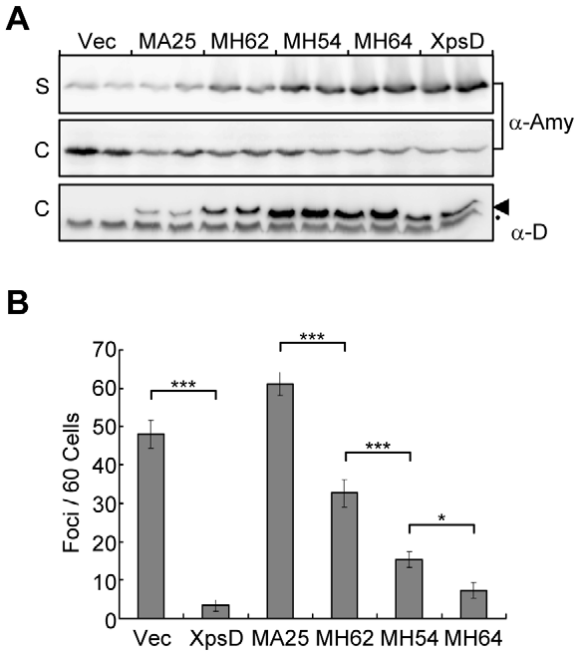
Inverse association of XpsE-ECFP foci abundance and secretion efficiency of the secretion channel. Plasmid-encoded XpsD::Myc mutant MA25, MH62, MH54 or MH64 was introduced into *xpsD* ^−^ strain with chromosome-encoded *xpsE-ecfp* (XC1753). The plasmid pAmy encoding the full-length α-amylase is present in all strains. (A) α-Amylase secretion analyzed by immunoblotting of culture supernatant (labeled as S) and cellular (labeled as C) fractions. Immunoblot analysis of the protein level of XpsD (arrowhead) in the cellular fraction (3^rd^ panel). The black dot (•) indicates the band crossreacting with anti-XpsD antiserum. Immunoblots for each strain are in duplicate. (B) Quantitative analysis of XpsE-ECFP foci abundance in strains supplemented with XpsD variants. Data are mean foci counts per 60 cells from 3 independent fields. * P = 0.058; *** P<0.001. Vec: empty vector; XpsD: plasmid-encoded wild-type XpsD.

Therefore, we used the linker insertion mutant *xpsD* acquired previously [22]. A Myc epitope was inserted at the original insertion site in *xpsD* (Table S2). The ability to restore the *xpsD*-null strain with α-amylase secretion could be ranked into 4 categories, ranging in degree of secretion recovery from null to complete, designated MA25, MH62, MH54 and MH64. XpsD(MA25) was apparently nonfunctional, but XpsD(MH64) was as functional as the wild-type XpsD (Figure 4A). XpsD(MH62) and the XpsD(MH54) were partially functional, with the former more defective. Fluorescence microscopy revealed that the abundance of focal appearing XpsE-ECFP in the strain containing different variants of XpsD::Myc was inversely associated with the degree of secretion recovery (Figures 4B and S3). The foci were most abundant in the strain containing the nonfunctional XpsD(MA25), at a level indistinguishable from that containing the empty vector. XpsD(MH62) and XpsD(MH54) showed an intermediate foci level, with more observed in the former strain, thus providing a well-defined inverse association of foci formation and secretion capacity of these strains. In the strain containing the functional XpsD(MH64), the foci were barely visible, similar to the strain containing the wild-type XpsD.

The protein abundance of the plasmid-encoded XpsD and its variants varied (Figure 4A). Of all variants examined, XpsD(MA25) was the least abundant. Yet, the chromosome-encoded XpsE-ECFP of the strain expressing the XpsD(MA25) showed the most distinct and abundant foci (Figures 4B and S3). Conversely, XpsD(MH64), present at a level higher than XpsD(MA25), did not support foci formation of XpsE-ECFP. These observations strongly argue against foci formation of XpsE-ECFP being a result of overexpression of the secretin.

### Restoration of normal function of the secretion channel paralleled stepwise reduction in abundance of XpsE-ECFP foci

**Figure 5:**
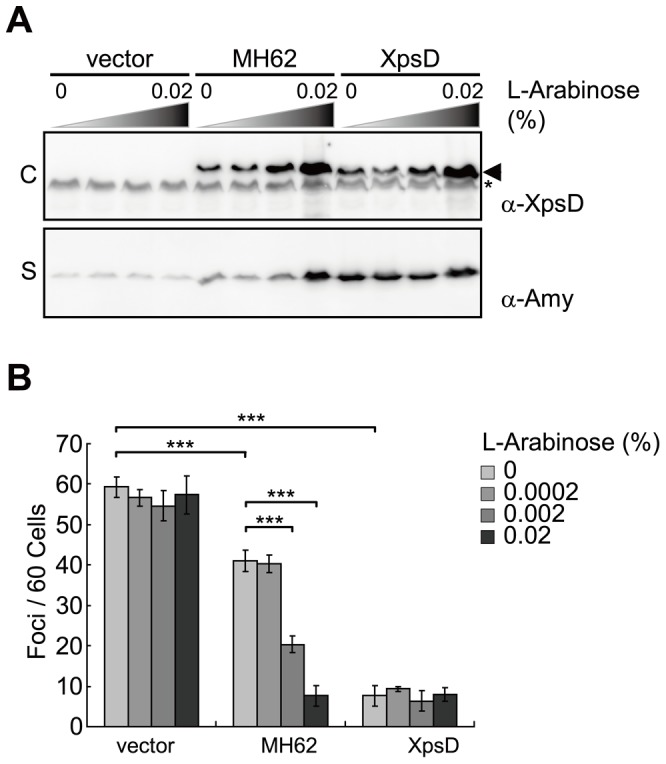
Reduced XpsE-ECFP foci abundance parallelled by secretion recovery in the secretion channel. (A) Immunoblot analysis of protein level of XpsD in cellular fraction (labeled as C) and secreted α-amylase in supernatant (labeled as S). The *xpsD* ^−^ mutant (XC1753) supplemented with the XpsD(MH62), wild-type XpsD (XpsD) or empty vector (vector) was grown in liquid media containing L-arabinose at increasing concentrations. Arrowhead indicates XpsD; *, cross-reactive band. (B) Quantitative analysis of XpsE-ECFP foci abundance in various strains grown in media with increasing concentrations of L-arabinose. Data are mean foci counts per 60 cells from 3 independent fields. *** P<0.001.

The secretion function of XpsD(MH62) was improved progressively by a stepwise increase in its protein abundance. As the level of secretin increased in response to increasing amount of the inducer L-arabinose, the level of secreted α-amylase increased in the culture medium (Figure 5A), accompanied by decreased number of XpsE-ECFP foci (Figure 5B and S4). The effect of L-arabinose was not observed in the cells containing the empty vector or the P_BAD_-driven wild-type XpsD. These observations are consistent with the focal-appearing XpsE-ECFP reverting to a diffused state with secretion through the T2S machine.

### Colocalization of XpsE-ECFP foci with the secretion channel

**Figure 6:**
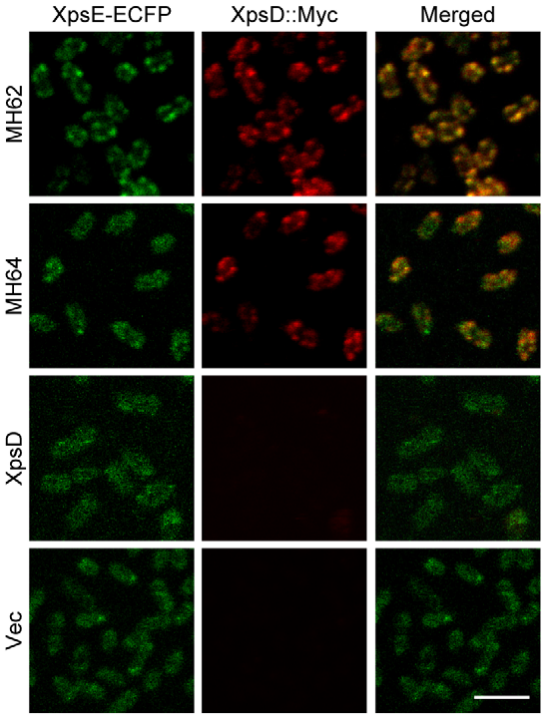
Colocalization of chromosome-encoded XpsE-ECFP foci with those of secretin by confocal microscopy. The *xpsD* ^−^ strain (XC1753) was supplemented with the partially functional XpsD(MH62) (the 1^st^ row) or secretion-proficient XpsD(MH64) (the 2^nd^ row) or the wild-type XpsD (the 3^rd^ row) or an empty vector (the 4^th^ row) and immunostained for XpsD::Myc with monoclonal antibody against Myc and fluorescence-labeled with Alexa Fluor 488. The XpsE-ECFP fluorescence is shown in green (left panels) and the immunostained XpsD::Myc in red (central panels). Shown in the last column are merged images (right panels). Scale bar, 2 µm.

To determine whether the focal-appearing XpsE-ECFP represents the ATPase convening at the T2S machine, we performed immunostaining of the Myc-tagged secretins XpsD(MH62) and XpsD(MH64) followed by confocal microscopy analysis. In the absence of L-arabinose, the former was partially functional and the latter was secretion-proficient. The plasmid encoding the wild-type XpsD devoid of Myc, as well as an empty vector, was also introduced into the *xpsD*-null strain and the cells examined in parallel. Consistent with the fluorescence microscopy of live cells, the XpsE-ECFP foci detected in the strain supplemented with the partially functional XpsD(MH62) were more abundant than the cells expressing the secretion-proficient XpsD(MH64) (Figure 6). The number of foci per cell detected by confocal microscopy appeared to be raised when compared with that detected by fluorescence microscopy. The increased foci could have arisen as a consequence of cell fixation while performing immunostaining. In addition, the images shown here were displayed with a magnification 2.5-times that of the fluorescence microscopy images. The fixation step and the high magnification may also explain why the XpsE-ECFP focused spots not as punctated and the cytoplasmic XpsE-ECFP not as evenly distributed as shown in the fluorescence microscopy images.

Immunostained fluorescence of both XpsD::Myc proteins appeared as focused spots at the cell periphery, albeit being more diffuse than the XpsE-ECFP foci shown in the fluorescence microscopy images. In addition, the number of XpsD::Myc foci per cell was much higher than that of the XpsE-ECFP foci shown in the fluorescence microscopy images. Both could be the result of cell fixation. As negative controls, the fluorescence of Alexa Fluor 488 was not detected in the absence of XpsD, and very faint in the presence of XpsD without the Myc tag. Consistent with the prediction, the XpsE-ECFP foci were less abundant in the strain supplemented with XpsD than in the strain supplemented with the empty vector.

The merged images revealed that XpsE-ECFP foci colocalized with those of both XpsD::Myc. However, colocalized spots were significantly more abundant in the strain expressing the partially functional XpsD(MH62) than in the secretion-proficient strain expressing the XpsD(MH64). Moreover, the XpsE-ECFP signal diffused in the cytoplasm was detectable only in the secretion-proficient strain, barely in the partially functional strain. These imply that the XpsE-ECFP appearing as focused spots is part of the T2S machine.

## Discussion

**Figure 7:**
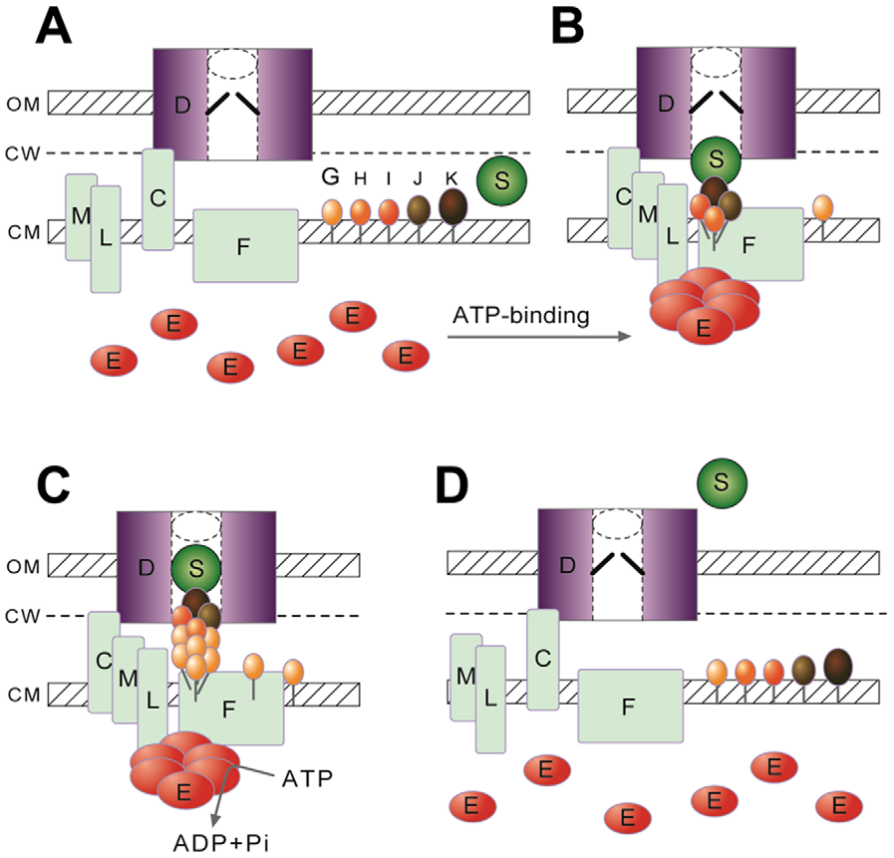
A proposed model of the type II secretion system depicting secretion-coupled positioning of the ATPase. (A) Before the exoprotein (designated S) reaches the secretion channel containing secretin (designated D), the ATPase (designated E) is present in cytoplasm as an ATP-free monomer. (B) As the exoprotein stalls at the entrance of the secretion channel, the ATPase, as an ATP-bound hexamer, convenes to the membrane-bound machine by direct interaction with the bitopic cytoplasmic membrane protein L. (C) ATP hydrolysis by the ATPase presumably drives assembly of pseudopilus from pseudopilins (designated G, H, I, J, K), which are primarily localized in cytoplasmic membrane or in association with the presumed ‘platform’ of F protein, to push the exoprotein through the gated channel. (D) As the exoprotein successfully passes through the secretion channel, the ATPase disperses from a membrane-bound state to cytoplasm. The dynamic status of cytoplasmic membrane proteins (F, L, M, C) and pseudopilins is speculative. OM, CW and CM, outer membrane, cell wall and cytoplasmic membrane, respectively.

In this study, we provide *in vivo* evidence showing that 1) the T2S ATPase working with the rest of the secretion machine is dynamic but well regulated and 2) the status of the exoprotein influences whether the ATPase associates with or dissociates from the machine. Thus, association of the T2S ATPase with the transenvelope multiprotein complex may be a pivotal step in the assembly of an active secretion machine (Figure 7). This proposition is consistent with *in vitro* studies showing that *V. cholerae* T2S ATPase activity was stimulated synergistically by the cytoplasmic domain of the membrane protein L and acidic phospholipid [18]. These results imply the active ATPase is able to drive pseudopilus assembly, as proposed for T4p extension, thus providing an outward-moving force to push exoprotein through the gated channel [5]. Alternatively, ATP hydrolysis by the machine-associated ATPase may, instead or additionally, be involved in pseudopilus disassembly for recycling pseudopilins, as was recently suggested for the ClpV1 ATPase in type VI secretion (T6S) [23]. In contrast to the T4p biogenesis, containing at least 2 ATPases, one for pilus assembly and the other for disassembly, only one ATPase has so far been identified in the T2S machines [24], [25].

Congruent with the idea that the T2S ATPase acts as a transiently associated part of the secretion machine, it was present in the secretion-proficient cells predominantly diffused in the cytoplasm. In this study, we could trap the short-living state as discrete foci at an elevated level by blocking the secretion channel and overproducing the exoprotein α-amylase. Proficient ATP binding is probably a prerequisite, because we could not observe the mutated XpsE(K331M, R504A) protein deficient in ATP binding at the cell periphery (Figure 3B). However, what triggers recruitment of the ATP-bound, and presumably oligomerized, ATPase remains to be determined. Before establishing stable association with the machine, the ATPase may associate with the cytoplasmic membrane momentarily. Liposome-binding of the *V. cholerae* T2S ATPase indicates that T2S ATPase may directly associate with the membrane [18]. The ATPase may retain an association with the membrane-bound machine and be engaged in secretion only when in close proximity to the secretion machine and the exoprotein is present in the periplasm.

The abundance of XpsE-ECFP focused spots decreased with stepwise recovery of normal function of the secretion channel (Figure 5B), which suggests that reduced exoprotein level in the periplasm may prompt dissociation of the membrane-bound XpsE-ECFP. When exoprotein secretion is complete, the transiently associated ATPase may be signaled to return to the cytoplasm (Figure 7). Our results did not reveal how the reduced exoprotein level in the periplasm is perceived by the ATPase. Attainment of pseudopilus assembly from pseudopilins may be the signal for completed secretion. Congruent with this idea, machine-association of the ATPase depends on the L protein (Figure 3A). As well, direct interaction between the L protein and the major pseudopilin of *V. cholerae* was recently demonstrated with *in vivo* crosslinking data [26]. The bitopic cytoplasmic membrane protein L would be eligible for detecting pseudopilus assembly and transmitting the signal across the membrane to the machine-associated ATPase, thus triggering its redistribution in cytoplasm. However, other components of the T2S machine may be involved. Our results can lead to further elaboration of these possibilities and lay foundations for deciphering how the T2S machine works as a multiprotein complex in coordination.

Fluorescence microscopy analysis of the *V. cholerae* T2S system indicated that secretin is required for 2 essential cytoplasmic membrane proteins, M and C, to form discrete foci at the cell boundary [27]. Thus, the outer membrane protein complex (i.e., the secretion channel) may support assembly of the cytoplasmic membrane protein complex, presumably of all 4 cytoplasmic membrane proteins [28]. In contrast, our observation of the L protein-dependent foci formation of the ATPase in absence of secretin implies that proper assembly of the cytoplasmic membrane protein complex is possible without secretin. Further studies are needed to reveal any differences in the biochemical or structural properties of the ATPase-containing complex assembled with or without secretin. Nevertheless, colocalization of the ATPase foci with those of the partially functional secretin XpsD(MH62) (Figure 6) is consistent with the idea that the ATPase foci we observed is a transiently associated part of the secretion machine.

The secretion function of the secretin variants we used appears to be dictated by the position of their Myc insertion. Sequence alignment of XpsD with other members of the secretin family revealed a modular N-terminal domain (residues 92–478) and a C-terminal secretin domain (residues 557–733) (Figure S5) [29]. Myc insertion in the nonfunctional XpsD(MA25) (*A645::Myc*) is located in the C-terminal secretin domain within a region defined as GATE 2 (residues 639–665) [29]. Deletion of GATE 2 in the secretin pIV required for filamentous phage assembly made the pIV protein nonfunctional in phage assembly without causing a major change in pIV multimer organization. In contrast, Myc inserted in the functional XpsD(MH64) (*A89::Myc*) and a partially functional XpsD(MH54) (*A186::Myc*) are located in the N-terminal domain of XpsD, one positioned upstream of the N0 module (residues 92–170) and the other in a loop upstream of the N1 module (residues 192–251) (Figure S5) [30]. Evidently, insertion at neither position caused major structural changes of the mutated secretin because the function of neither was greatly affected. Finally, Myc insertion in the partially functional XpsD(MH62) (*A380::Myc*) is in the N3 module within a region rich in glycine and serine in XpsD. Fitting the 12-member ring model of the *V. cholerae* secretin EpsD N-terminal domain (N0–N3) into the EpsD density map acquired from cryoEM reconstruction revealed that the N3 module fits into the constriction in the middle of the cavity at the entrance end of the secretion channel [4]. With increased secretion efficiency of XpsD(MH62) by its overproduction, the Myc insertion site of this XpsD variant is probably positioned in a structurally flexible region of the secretin causing defects in the secretion channel that can be partially overcome by increasing abundance of the variant secretin.

As a cytoplasmic component of the T2S machine, ATPase apparently transiently joins the remaining parts of the transenvelope machine to engage in active secretion. In contrast, the XpsE homologue VirB11 of the *Agrobacterium tumefaciens* type IV secretion (T4S) machine appeared solely as discrete foci at the cell periphery, as revealed by immunofluorescence microscopy [31]. The VirB11 distribution pattern is similar to that of the components of the T4S core complex, VirB7, VirB9 and VirB10 [32], so the T4S ATPase VirB11 may be a stable part of the secretion machine. Although not so closely related to the T2S ATPase, the T6S ATPase ClpV1 also acts as an essential soluble component of the secretion machine. Punctate localization of the ClpV1-GFP was observed in wild-type *Pseudomonas aeruginosa*, favoring its being a fixed part of the secretion machine [33]. However, ClpV1 was recruited to the secretion machine in a phosphorylation-dependent manner [34], [35]. Because the cue for activating phosphorylation is unknown, ClpV1 may behave dynamically in accordance with the secretion activity as well.

The T2S system is closely related to the T4p biogenesis system by possessing 5 pseudopilins, similar to the subunit of T4p. Additional homologous counterparts of the T2S machine (i.e., secretin, a polytopic cytoplasmic membrane protein and ATPase) are indispensable for T4p biogenesis [36]. Moreover, the major pseudopilin G assembles into a filamentous structure similar to that of T4p [8]–[10]. T4p ATPases may be recruited to the machine in a transient manner as well. In particular, the 2 ATPases of the T4p biogenesis machine act antagonistically, one for pilus extension and the other for retraction [37]. One way to prevent the 2 ATPases from counteracting each other would be for each to be recruited to the machine only when its function is required and/or released as soon as the function is fulfilled. Of all the T4p ATPases examined by fluorescent protein tagging, only discrete foci were observed [38], [39]. However, dynamic localization of the T4p ATPases was demonstrated in *Myxococcus xanthus*, whose T4p are unipolarly localized and undergo pole-to-pole oscillations [40]. Three core proteins of T4p machine localize in a bipolar, symmetric pattern and remain stationary at the poles during reversals. In contrast, the PilB and PilT motor ATPases, which energize extension and retraction, respectively, localize at opposite poles, with PilB predominantly at the piliated pole and PilT predominantly at the non-piliated pole. The retraction ATPase PilT relocates between poles during reversals, which suggests that the 2 evolutionarily related machines share an additional common feature by their ATPase being a transiently associated part of the active machine.

Our observations raise an intriguing possibility regarding the operational mechanism of the T2S machine. Secretion-related dynamic behavior may be a central theme of the secretion machine. Components other than the ATPase may also be assembled transiently. For instance, the pseudopilus elongation from the major pseudopilin may not be initiated until the ATPase is activated. Time-lapse microscopy may be required for visualizing such a dynamic event. Alternatively, biochemical approaches, such as position-specific crosslinking of the major pseudopilin, could be used to monitor the assembled pseudopilus [8].

## Materials and Methods

### Bacterial strains and plasmid construction

All strains used in this study are derivatives of *X. campestris* pv campestris XC1701, a spontaneously arising rifampicin-resistant mutant of a natural isolate, XC17 [20]. The relevant characteristics are in Supporting Information Table S1. Chromosome integration and modification involved the *sacB*-based two-step selection procedure [19]. The strains with chromosome-encoded XpsE-ECFP, XC1751 (*xpsD*-plus strain with integrated *xpsE-ecfp*), XC1753 (*xpsD*-null with integrated *xpsE-ecfp*) and XC1760 (*xpsD*/*xpsL* double knockout with integrated *xpsE-ecfp*), were generated by using the suicidal plasmid pUCDECstF to replace *xpsE* with *xpsE-ecfp* in XC1701, XC1708 and XC1741 (*xpsD*/*xpsL* double knockout), respectively. XC1741 was generated by replacing the full-length *xpsD* gene in XC1712 with the deleted *xpsD* gene by using pUCWD. Chromosome integrated *xpsE-ecfp* and the deleted alleles were confirmed by PCR. The strain XC1757, with integrated *xpsE(K331M, R504A)-ecfp* in an *xpsD*-plus background, was generated by introducing the mutations K331M and R504A one-by-one into XC1751 by using pUCD-E(KM)CstF and pUCD-E(KMRA)CstF sequentially. Similarly, the strain XC1758, with integrated *xpsE(K331M, R504A)-ecfp* in an *xpsD*-null background, was generated by introducing the mutations K331M and R504A one-by-one into XC1753. Mutations in the *xpsE* gene were confirmed by sequencing the PCR-amplified DNA fragment from the chromosomal *xpsE-ecfp* gene.

All plasmids are listed in Supporting Information Table S2. DNA manipulation involved the standard molecular cloning protocol. The *ecfp* gene fused to the 3′ end of the *xpsE* gene was carried on a broad host-range plasmid pCPP30 or integrated into the chromosome replacing the *xpsE* gene as described previously. The *xpsD::Myc* variants carried on pCPP30 are derived from the linker insertion mutant *xpsD* genes constructed previously [22]. Their function in supporting T2S was determined by assaying their ability to complement the *xpsD*-null strain in α-amylase secretion on a starch plate [22].

### Immunoblot analysis

One milliliter of *X. campestris* pv. campestris was harvested at 1 OD_600_, washed with water and resuspended in 100 µl sample buffer as a cellular fraction. Four-hundred microliters of the supernatant precipitated with ice-cold acetone was resuspended in 40 µl sample buffer and collected as an extracellular fraction. Proteins in each fraction were separated on SDS-polyacrylamide gel (10% or 12% acrylamide), then electroblotted onto polyvinylidene difluoride membrane. After being blocked with 6% skim milk in TBS buffer (30 mM Tris-HCl, pH 7.4, 250 mM NaCl), the membrane was incubated with antiserum against the target protein, then with horseradish peroxidase-conjugated goat anti-rabbit IgG (or goat anti-mouse IgG). The target protein on the membrane was visualized by incubating with a Western Lightening® Chemiluminescence Reagent *Plus* (PerkinElmer) mixture and detected by use of the LAS-3000 mini (Fujifilm) image reader. Rabbit antisera against α-amylase, XpsE, XpsL and XpsD were obtained from previous studies [17], [41]–[43]. Rabbit antiserum against GFP was purchased from Invitrogen. Mouse monoclonal anti-c-Myc antibody was from Roche.

### Fluorescence microscopy

Fluorescence microscopy involved use of an Olympus BX51 microscope equipped with fluorescence filter sets, U-MCFPHQ and U-MWG2, and an NA 1.3/100× oil immersion objective lens. ECFP fusion in live cells was visualized. Cells grown in LB broth to A_600_ 1 were washed once with HBSS buffer (Invitrogen), stained for 3 min with the vital membrane dye FM4-64 (Invitrogen; 1∶2000 dilution) and placed on HBSS-buffered 1% agarose pads on glass slides. Fluorescent images were recorded by use of a DP71 CCD camera coupled with DP Controller software v3.1.1.267 (Olympus). Images were processed by use of DP Manager v3.1.1.208 (Olympus) and Adobe Photoshop CS3.

### Immunofluorescence staining

Bacteria grown to A_600_ 1 were collected, washed once with phosphate buffered saline (PBS) and resuspended in one-fifth volume of PBS. Cells immobilized on L-poly-lysine–treated coverslips (GE) were fixed for 20 min with 4% para-formaldehyde in PBS. Coverslips were then washed with PBS and immersed in blocking buffer (PBS containing 10% fetus bovine serum, 0.2% Triton X-100) for 30 min. The blocking solution was replaced with hybridization solution (PBS containing 5% fetus bovine serum, 0.2% Triton X-100) containing anti-cMyc monoclonal antibody (Roche) (1∶250). After 2 h, coverslips were washed 4 times with 1 ml 0.2% Triton X-100-containing PBS and incubated at room temperature for 1 h with 250 µl hybridization solution containing goat anti-mouse IgG conjugated with Alexa Fluor 488 (Invitrogen; 1∶250) avoiding light. The following process was kept from light: 4 times washing with 1 ml 0.2% Triton X100-containing PBS solution, cell mounting in ProLong® Gold antifade reagent (Invitrogen) and sealing of coverslips on a glass microscope slide with nail polish.

### Laser scanning confocal microscopy

Fluorescence emitted by Alexa Fluor 488-stained XpsD::Myc and chromosome-encoded XpsE-ECFP was captured by use of confocal microscopy (FluoView FV1000, Olympus) with an NA 1.4/100× oil immersion objective lens. Alexa Fluor 488 was excited at 488 nm laser and emitted signals were detected from 500 to 530 nm through a PMT detector by sequential line scanning. For endogenous XpsE-ECFP imaging, ECFP was excited with an LD 440 laser (440 nm UV) and emitted signals were detected from 460 to 560 nm through a PMT detector by sequential line scanning. Ratiometric analysis and merging of the images involved use of FluoView FV1000 software FV10-ASW 3.0 (Olympus). Images were assembled by use of Adobe Photoshop 6.0.

### Statistical analysis

The number of fluorescent foci detected in 60 cells was quantified in 3 fields, each with approximately 60 cells, in the phase contrast images. Statistical analysis involved one-way ANOVA using SPSS software v13.

## Supporting Information

Figure S1 **Functional assay of the chromosome-encoded XpsE-ECFP and protein level analysis.** (**A**) α-Amylase secretion was assayed on starch plates. The parental strain (*xpsE*, *xpsD* ^+^) and *xpsD*-null strain (*xpsE*, *xpsD* ^−^) are positive and negative controls, respectively. *xpsE-ecfp* represents the integrated *xpsE-ecfp* gene that has replaced the chromosomal *xpsE* gene. (**B**) Immunoblot analysis of protein level of chromosome-encoded XpsE-ECFP (arrow next to the top panel). Arrowhead indicates the XpsE protein. The bottom panel confirms the strains as *xpsD*-plus or *xpsD*-null. The *xps* ^−^ strain, which is missing the entire *xps* gene cluster, is a negative control. * and *** indicate the cross-reactive band in the *X. campestris* pv. campestris cell lysates interacting with anti-XpsE and anti-XpsD antiserum, respectively.
(TIF)

Figure S2 **Specific enhancement of the chromosome-encoded XpsE-ECFP foci intensity and abundance in the** ***xpsD*** **-null strain by overproducing α-amylase.** (**A**) Fluorescence microscopy of chromosome-encoded XpsE-ECFP in the *xpsD*-plus (top panels) or *xpsD*-null background (bottom panels) supplemented with an empty vector (Vec), plasmid-encoded truncated α-amylase missing its N-terminal signal peptide (cAmy), plasmid-encoded full-length α-amylase (Amy), or plasmid-encoded maltose binding protein (MBP). Scale bar, 5 µm. (**B**) Quantitative analysis of foci abundance shown in (A). Data are mean foci counts per 60 cells from 3 independent fields. ***, P<0.001. (**C**) Immunoblot analysis of distribution of α-amylase (top 2 panels) or MBP (3^rd^ and 4^th^ panel) in culture supernatant (S) or cellular fraction (C). Bottom 2 panels show strain confirmation (cellular fraction interacting with anti-XpsD antiserum) and protein abundance of XpsE-ECFP (cellular fraction interacting with anti-XpsE antiserum). Black dot (•) indicates cross-reactive band in *X. campestris* pv. campestris cell lysates interacting with anti-XpsD antiserum.
(TIF)

Figure S3 **Fluorescence microscopy of chromosome-encoded XpsE-ECFP in the** ***xpsD*** **-null strain supplemented with various** ***xpsD::myc*** **mutants.** Plasmid-encoded XpsD::Myc mutant MA25, MH62, MH54 or MH64 was introduced into the *xpsD* ^−^ strain with chromosome-encoded *xpsE-ecfp* gene (XC1753). The plasmid pAmy encoding the full-length α-amylase is present in all strains. Vec: empty vector; XpsD: plasmid-encoded wild-type XpsD. Scale bar, 5 µm.
(TIF)

Figure S4 **Fluorescence microscopy of chromosome-encoded XpsE-ECFP in the** ***xpsD*** **-null strain supplemented with the P_BAD_-driven** ***xpsD::myc*** **mutant MH62 carried on a broad host range vector and grown with increasing concentrations of L-arabinose.** The wild-type XpsD (top panels) and empty vector (bottom panels) are included for comparison. The plasmid pAmy encoding the full-length α-amylase is present in all 3 strains. Scale bar, 10 µm.
(TIF)

Figure S5 **Domain and modular organization of XpsD.** Multiple sequence alignment of XpsD and its homologues involved use of Clustal W v1.81. Aligned sequences include 1) the pIV protein of filamentous phage f1 (NCBI, P03666), 2) OutD of *Dickeya dadantii* (formerly *Erwinia chrysanthemi*) (NCBI, CAA46370), 3) PulD of *Klebsiella oxytoca* (NCBI, AAA25126), 4) XcpQ of *Pseudomonas aeruginosa* (NCBI, CAA48582), 5) XpsD of *X. campestris* pv. campestris (NCBI, AAA27615) and 6) the N-terminal domain of the ETEC GspD of enterotoxigenic *Escherichia coli* (NCBI, AAL10693, residues 1–241). The regions defined as the secretin domain and N1, N2 modules were assigned by NCBI. Assignment of the N0 and N3 modules were based on sequence alignment with ETEC GspD. Assignment of the regions defined as GATE 1 and GATE 2 (depicted as white boxes in the secretin domain) were based on sequence alignment with pIV. ‘GS-rich’ (depicted as white box in the N3 module) designates the region of XpsD rich in glycine and serine. The N-terminal signal sequence is in black and the conserved modules and domain are in grey.
(TIF)

Table S1 **Bacterial Strains.**
(DOC)

Table S2 **Plasmids.**
(DOC)
