# Supplementary figures and images for: Function-Related Positioning of the Type II Secretion ATPase of Xanthomonas campestris pv. campestris
Source: PLoS One. 2013 Mar 11;8(3):e59123. doi: 10.1371/journal.pone.0059123 (PMC3594185; doi:10.1371/journal.pone.0059123)

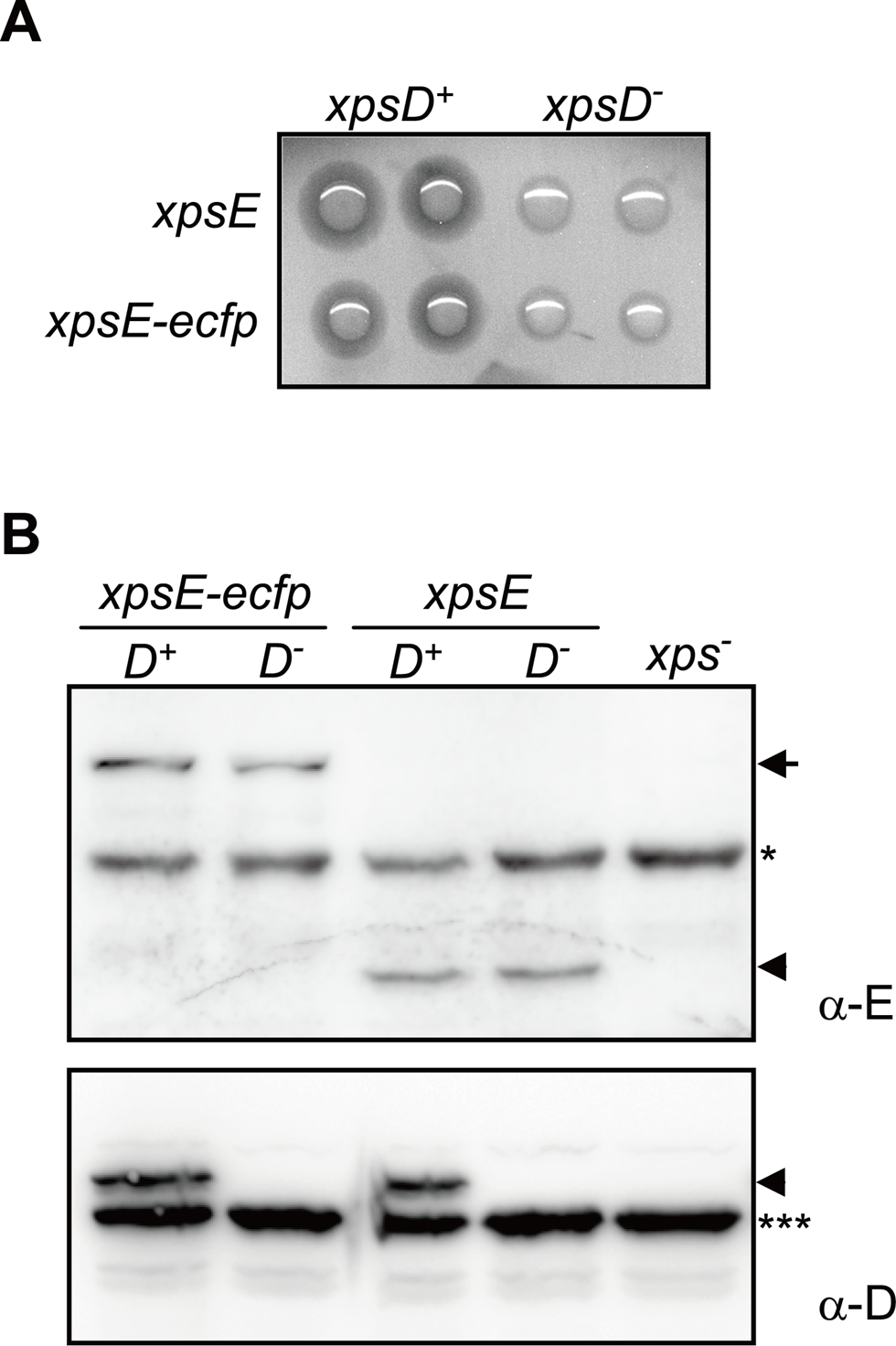

Supplement: Figure S1 — Functional assay of the chromosome-encoded XpsE-ECFP and protein level analysis. (A) α-Amylase secretion was assayed on starch plates. The parental strain (xpsE, xpsD +) and xpsD-null strain (xpsE, xpsD −) are positive and negative controls, respectively. xpsE-ecfp represents the integrated xpsE-ecfp gene that has replaced the chromosomal xpsE gene. (B) Immunoblot analysis of protein level of chromosome-encoded XpsE-ECFP (arrow next to the top panel). Arrowhead indicates the XpsE protein. The bottom panel confirms the strains as xpsD-plus or xpsD-null. The xps − strain, which is missing the entire xps gene cluster, is a negative control. * and *** indicate the cross-reactive band in the X. campestris pv. campestris cell lysates interacting with anti-XpsE and anti-XpsD antiserum, respectively. (TIF) [file pone.0059123.s001.tif]

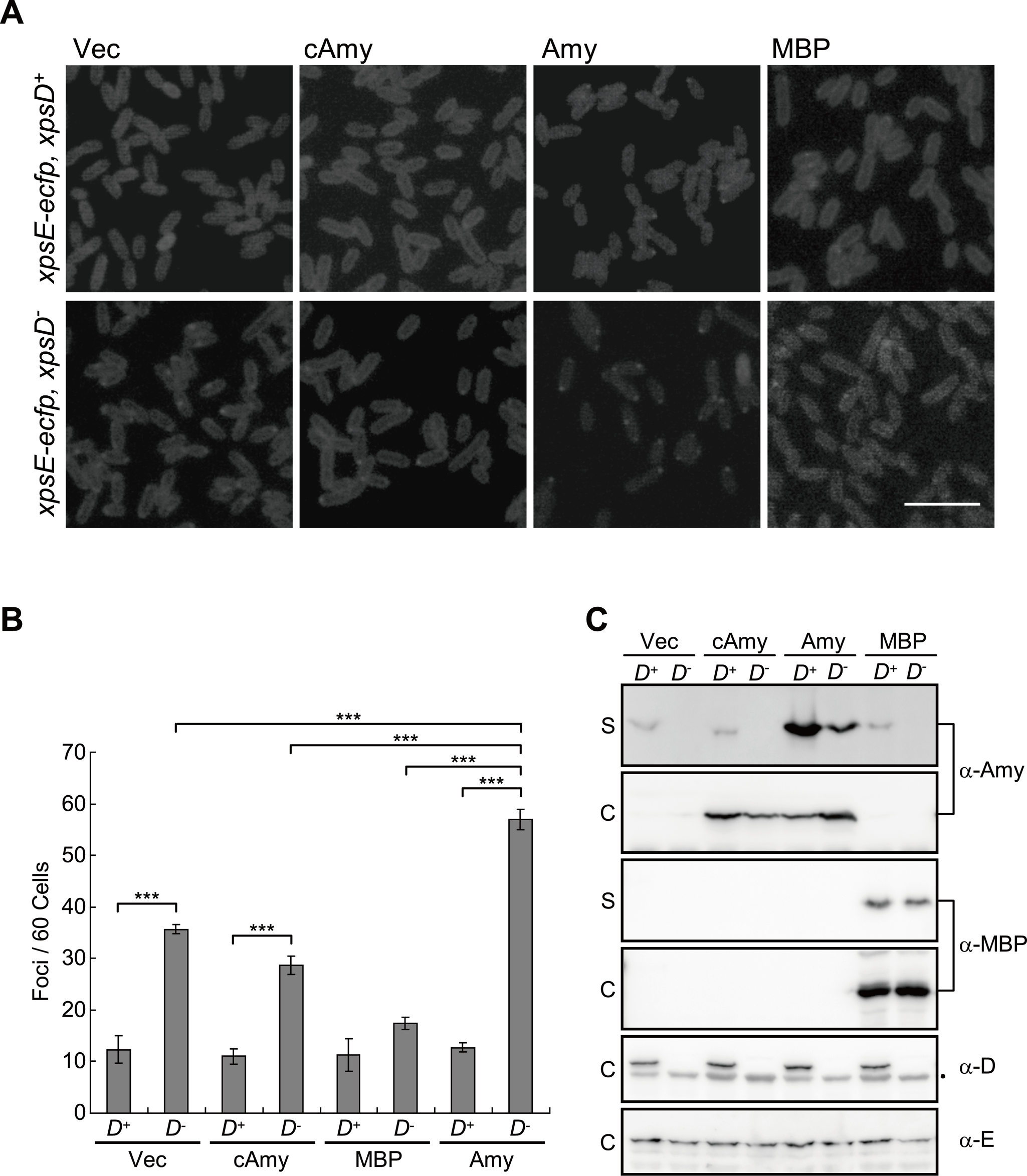

Supplement: Figure S2 — Specific enhancement of the chromosome-encoded XpsE-ECFP foci intensity and abundance in the xpsD -null strain by overproducing α-amylase. (A) Fluorescence microscopy of chromosome-encoded XpsE-ECFP in the xpsD-plus (top panels) or xpsD-null background (bottom panels) supplemented with an empty vector (Vec), plasmid-encoded truncated α-amylase missing its N-terminal signal peptide (cAmy), plasmid-encoded full-length α-amylase (Amy), or plasmid-encoded maltose binding protein (MBP). Scale bar, 5 µm. (B) Quantitative analysis of foci abundance shown in (A). Data are mean foci counts per 60 cells from 3 independent fields. ***, P<0.001. (C) Immunoblot analysis of distribution of α-amylase (top 2 panels) or MBP (3rd and 4th panel) in culture supernatant (S) or cellular fraction (C). Bottom 2 panels show strain confirmation (cellular fraction interacting with anti-XpsD antiserum) and protein abundance of XpsE-ECFP (cellular fraction interacting with anti-XpsE antiserum). Black dot (•) indicates cross-reactive band in X. campestris pv. campestris cell lysates interacting with anti-XpsD antiserum. (TIF) [file pone.0059123.s002.tif]

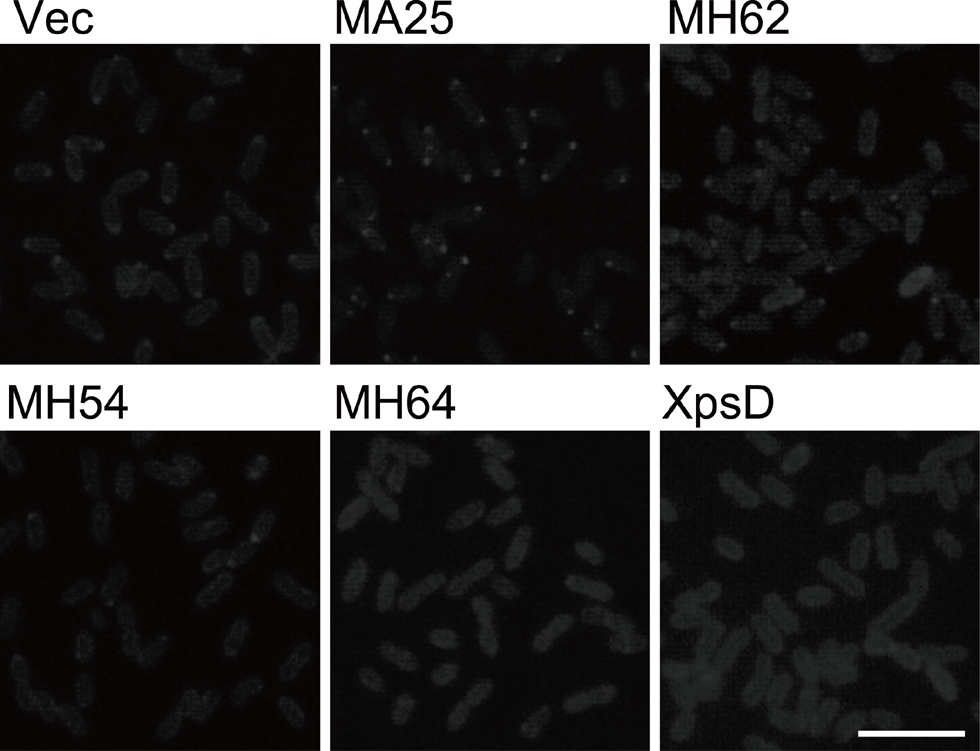

Supplement: Figure S3 — Fluorescence microscopy of chromosome-encoded XpsE-ECFP in the xpsD -null strain supplemented with various xpsD::myc mutants. Plasmid-encoded XpsD::Myc mutant MA25, MH62, MH54 or MH64 was introduced into the xpsD − strain with chromosome-encoded xpsE-ecfp gene (XC1753). The plasmid pAmy encoding the full-length α-amylase is present in all strains. Vec: empty vector; XpsD: plasmid-encoded wild-type XpsD. Scale bar, 5 µm. (TIF) [file pone.0059123.s003.tif]

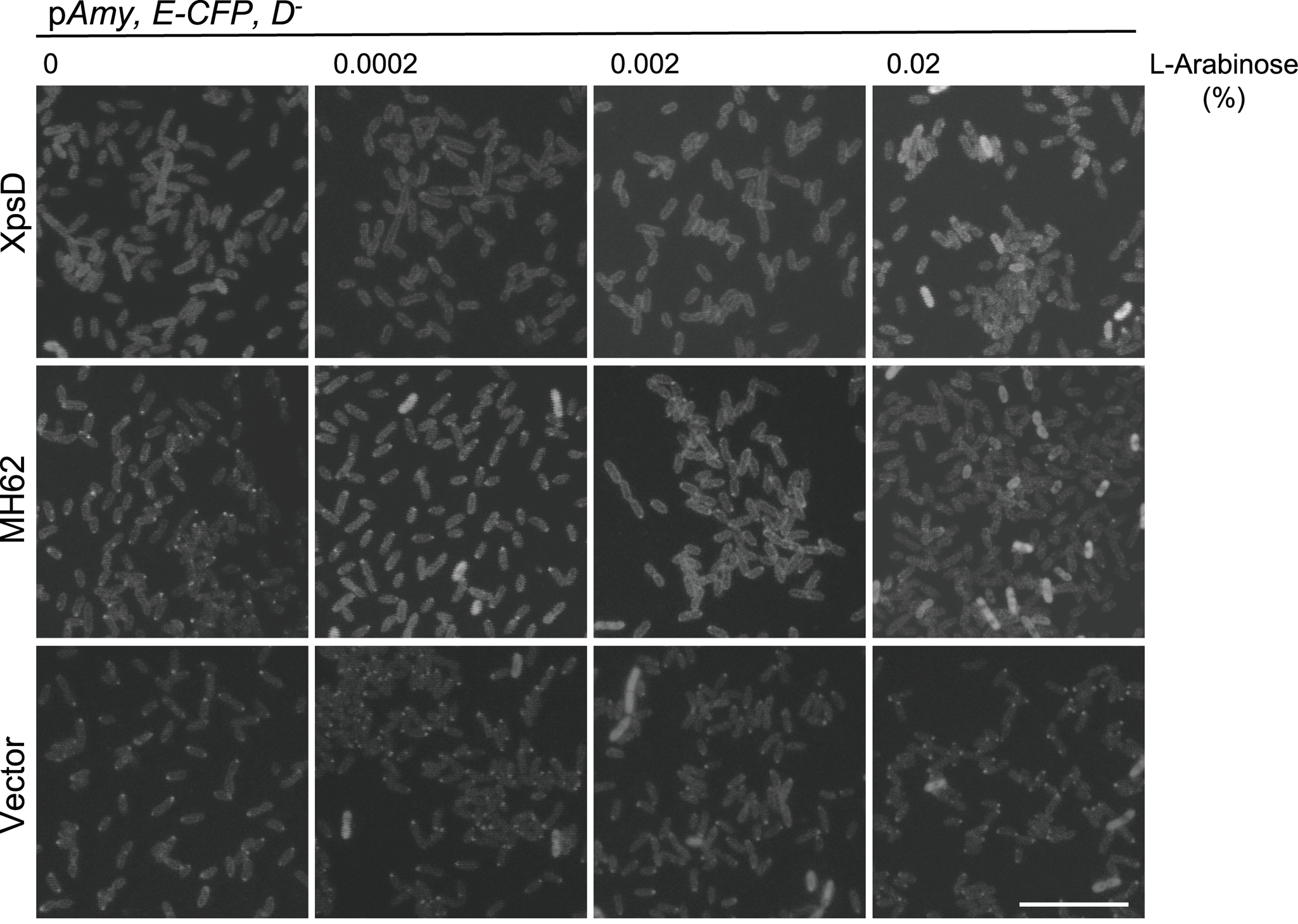

Supplement: Figure S4 — Fluorescence microscopy of chromosome-encoded XpsE-ECFP in the xpsD -null strain supplemented with the PBAD-driven xpsD::myc mutant MH62 carried on a broad host range vector and grown with increasing concentrations of L-arabinose. The wild-type XpsD (top panels) and empty vector (bottom panels) are included for comparison. The plasmid pAmy encoding the full-length α-amylase is present in all 3 strains. Scale bar, 10 µm. (TIF) [file pone.0059123.s004.tif]

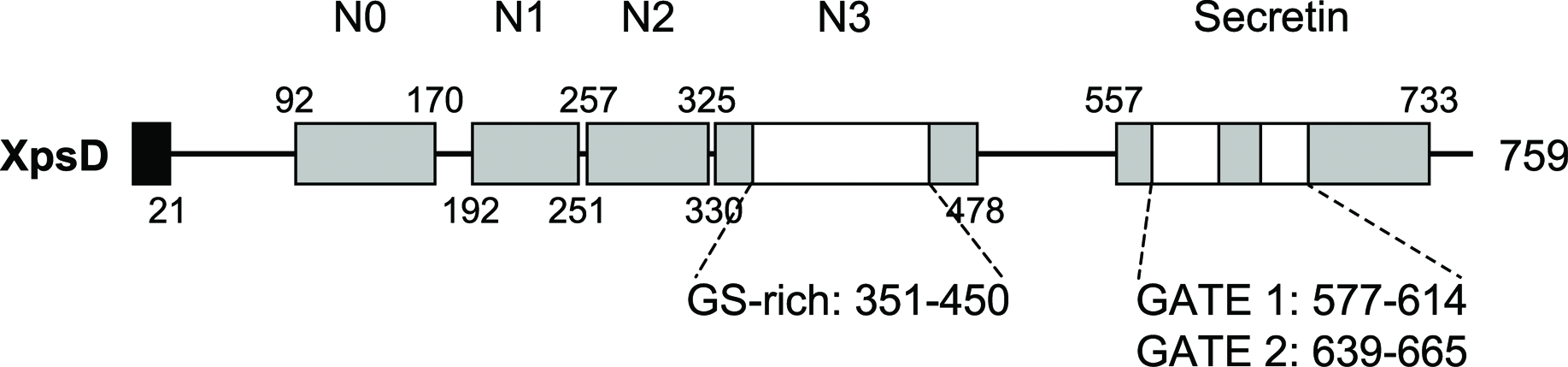

Supplement: Figure S5 — Domain and modular organization of XpsD. Multiple sequence alignment of XpsD and its homologues involved use of Clustal W v1.81. Aligned sequences include 1) the pIV protein of filamentous phage f1 (NCBI, P03666), 2) OutD of Dickeya dadantii (formerly Erwinia chrysanthemi) (NCBI, CAA46370), 3) PulD of Klebsiella oxytoca (NCBI, AAA25126), 4) XcpQ of Pseudomonas aeruginosa (NCBI, CAA48582), 5) XpsD of X. campestris pv. campestris (NCBI, AAA27615) and 6) the N-terminal domain of the ETEC GspD of enterotoxigenic Escherichia coli (NCBI, AAL10693, residues 1–241). The regions defined as the secretin domain and N1, N2 modules were assigned by NCBI. Assignment of the N0 and N3 modules were based on sequence alignment with ETEC GspD. Assignment of the regions defined as GATE 1 and GATE 2 (depicted as white boxes in the secretin domain) were based on sequence alignment with pIV. ‘GS-rich’ (depicted as white box in the N3 module) designates the region of XpsD rich in glycine and serine. The N-terminal signal sequence is in black and the conserved modules and domain are in grey. (TIF) [file pone.0059123.s005.tif]
