# Supplementary material for: Function-Related Positioning of the Type II Secretion ATPase of Xanthomonas campestris pv. campestris
Source: PLoS One. 2013 Mar 11;8(3):e59123. doi: 10.1371/journal.pone.0059123 (PMC3594185; doi:10.1371/journal.pone.0059123)
Supplement: Table S1 — Bacterial Strains. (DOC) [file pone.0059123.s006.doc]

**Table S1. Bacterial strains**

| **Species** | **Strain** | **Relevant Genotype** | **Reference/Source** |
| --- | --- | --- | --- |
|  |  |  |  |
| *X. campertris* pv. campestris | XC1701 | Rif^r^, *xps^+^* | [1] |
|  | XC1723 | Rif^r^, Δ*xpsE* | [2] |
|  | XC1708 | Rif^r^, *xpsD*::Tn*5* | [1] |
|  | XC17433 | Rif^r^, *xps^-^* ( XC1701::Tn*5*) | [1] |
|  | XC1751 | Rif^r^, *xpsE-ecfp* | This study |
|  | XC1753 | Rif^r^, *xpsE-ecfp, xpsD*::Tn*5* | This study |
|  | XC1757 | Rif^r^, *xpsE(K331M, R504A)-ecfp* | This study |
|  | XC1758 | Rif^r^, *xpsE(K331M, R504A)-ecfp*, *xpsD*::Tn*5* | This study |
|  | XC1741 | Rif^r^, Δ*xpsL,* Δ*xpsD* | This study |
|  | XC1760 | Rif^r^, *xpsE-ecfp,* Δ*xpsL,* Δ*xpsD* | This study |
| *E. coli* | DH5α | F^-^ *supE*44 Δ*lacU169* (φ80 *lacZ*Δ*M15*) *hsdR17 recA*1 *endA*1 *gyrA*96 *thi*-1 *relA*1 | [3] |
|  | GM48 | F^-^ *dam-*3 *dcm-*6 *thr-*1 *leuB*6 *ara-*14 *tonA*31 *lacY*1 *tsx-*78 *supE*44 *galK*2 *galT*22 *thi-*1 | [4] |
|  |  |  |  |
