# Supplementary material for: Function-Related Positioning of the Type II Secretion ATPase of Xanthomonas campestris pv. campestris
Source: PLoS One. 2013 Mar 11;8(3):e59123. doi: 10.1371/journal.pone.0059123 (PMC3594185; doi:10.1371/journal.pone.0059123)
Supplement: Table S2 — Plasmids. (DOC) [file pone.0059123.s007.doc]

**Table S2. Plasmids**

| **Purpose** | **Plasmid Name** | **Relevant Description** | **Source** |
| --- | --- | --- | --- |
|  |  |  |  |
| *For complementation* | pCPP30 | a broad-host-range cloning vector, IncP, Tc^r^ | Bauer, David |
|  | pCW2 | *xpsE-Strep*, *xpsF*, IncP, Tc^r^ | This study |
|  | pCW3 | cwflinker2, *xpsF*, IncP, Tc^r^ | This study |
|  | pcwECFP | *ecfp,* IncP, Tc^r^ | This study |
|  | pcwEC7 | *xpsE-ecfp*, IncP, Tc^r^ | This study |
|  | pL2 | abbreviation of pCPP30L2; *xpsL*, IncP, Tc^r^ | [5] |
| *For substrate-specific enhancement of XpsE-ECFP foci formation* | pAmy | *amy,* *ori*pBBR1, Gm^r^ | This study |
|  | pcAmy | *c-amy,* *ori*pBBR1, Gm^r^ | This study |
|  | pMBP | *malE,* *ori*pBBR1, Gm^r^ | This study |
| *For secretin manipulation* | pBBR1MCS5 | a broad-host-range cloning vector which is compatible with IncP, IncQ, and IncW group plasmids, as well as with ColE1- and P15a-based replicons, Gm^r^ | [6] |
|  | pBAD18 | a pBAD vector containing the P_BAD_ promoter of the arabinose operon and its regulatory gene *araC*, Ap^r^ | [7] |
|  | pHMBAD2 | BAD regulon containing P_BAD_ promoter and its regulatory gene *araC* derived from pBAD18 subcloned in pBBR1MCS5, Gm^r^ | This study |
|  | pB2D | P_BAD_-*xpsD*, *araC*, *ori*pBBR1, Gm^r^ | This study |
|  | pB2MA25 | P_BAD_*-xpsD(A645::Myc)*, *araC*, *ori*pBBR1, Gm^r^ | This study |
|  | pB2MH62 | P_BAD_*-xpsD(A380::Myc)*, *araC*, *ori*pBBR1, Gm^r^ | This study |
|  | pB2MH54 | P_BAD_*-xpsD(A186::Myc)*, *araC*, *ori*pBBR1, Gm^r^ | This study |
|  | pB2MH64 | P_BAD_*-xpsD(A89::Myc)*, *araC*, *ori*pBBR1, Gm^r^ | This study |
|  | pcwBAD2 | BAD regulon containing P_BAD_ promoter and its regulatory gene *araC* derived from pBAD18 subcloned in pCPP30, Tc^r^ | This study |
|  | pcwB2D | P_BAD_-*xpsD*, *araC*, IncP, Tc^r^ | This study |
|  | pcwB2MA25 | P_BAD_*-xpsD(A645::Myc)*, *araC*, IncP, Tc^r^ | This study |
|  | pcwB2MH62 | P_BAD_*-xpsD(A380::Myc)*, *araC*, IncP, Tc^r^ | This study |
|  | pcwB2MH54 | P_BAD_*-xpsD(A186::Myc)*, *araC*, IncP, Tc^r^ | This study |
|  | pcwB2MH64 | P_BAD_*-xpsD(A89::Myc)*, *araC*, IncP, Tc^r^ | This study |
| *For strain construction* | pUCD4121 | *sacB, ori*pUC, Cm^r^ | [8] |
|  | pLBPU | *ΔxpsL*-flanks, *sacB*, *ori*pUC, Cm^r^, Km^r^ | Leu, W-M |
|  | pUCWD | *ΔxpsD*-flanks, *sacB*, *ori*pUC, Cm^r^, Km^r^ | This study |
|  | pUCDLnkG | *XbXh-linker*, *sacB*, *ori*pUC, Cm^r^, Gm^r^ | This study |
|  | pUCD-ECst-F | *xpsE-ecfp-Strep-xpsF*, *sacB*, *ori*pUC, Cm^r^, Gm^r^ | This study |
|  | pUCD-E(KM)Cst-F | *xpsE(KM)-ecfp-Strep-xpsF*, *sacB*, *ori*pUC, Cm^r^, Gm^r^ | This study |
|  | pUCD-E(KMRA)Cst-F | *xpsE(KMRA)-ecfp-Strep-xpsF*, *sacB*, *ori*pUC, Cm^r^, Gm^r^ | This study |
|  |  |  |  |
